# Impact of tooth loss related to number and position on oral health quality of life among adults

**DOI:** 10.1186/s12955-014-0165-5

**Published:** 2014-11-30

**Authors:** Marília Jesus Batista, Herenia Procopio Lawrence, Maria da Luz Rosário de Sousa

**Affiliations:** Department of Community Dentistry, Piracicaba Dental School, University of Campinas, Avenida Limeira, 901, P.O. Box 52, Piracicaba, 13414-018 SP Brazil; Dental Public Health Discipline, Faculty of Dentistry, University of Toronto, Canada, 124 Edward Street, Toronto, M5G 1G6 Ontario Canada

**Keywords:** Adults, Oral health, Quality of life, OHIP14, Regression analysis

## Abstract

**Background:**

The objective of this study was to evaluate the impact of tooth loss on oral health-related quality of life (OHRQoL) in adults with emphasis on the number of teeth lost and their relative position in the mouth.

**Methods:**

The study population was a cross-sectional household probability sample of 248, representing 149,635 20–64 year-old residents in Piracicaba-SP, Brazil. OHRQoL was measured using the OHIP-14. Socioeconomic, demographic, health literacy, dental services use data and clinical variables were collected. Oral examinations were performed using WHO criteria for caries diagnosis, using the DMFT index; that is, the sum of decayed, missing and filled teeth (DMFT). An ordinal scale for tooth loss, based on position and number of missing teeth, was the main explanatory variable. The total OHIP score was the outcome for negative binomial regression and OHIP prevalence was the outcome for logistic regression at 5% level. A hierarchical modeling approach was adopted according to conceptual model.

**Results:**

OHIP score was 10.21 (SE 1.16) with 48.1% (n=115) reporting one or more impacts fairly/very often (OHIP prevalence). Significant prevalence rate ratios (PRRs) for OHIP severity were observed for those who had lost up to 12 teeth, including one or more anterior teeth (PRR=1.63, 95%CI 1.06–2.51), those who had lost 13–31 teeth (PRR=2.33, 95%CI 1.49–3.63), and the edentulous (PRR=2.66, 95%CI 1.55–4.57) compared with fully dentate adults. Other significant indicators included those who only sought dental care because of dental pain (PRR=1.67, 95%CI 1.11–2.51) or dental needs (PRR=1.84, 95%CI 1.24–2.71) and having untreated caries (PRR=1.57 95%CI 1.09–2.26). Tooth loss was not significantly associated with OHIP prevalence; instead using dental services due to dental pain (PR=2.43, 95%CI 1.01–5.82), having untreated caries (PR=3.96, 95%CI 1.85–8.51) and low income (PR=2.80, 95%CI 1.26–6.42) were significant risk indicators for reporting OHIP prevalence.

**Conclusion:**

Our analyses showed OHRQoL gradients consistent with the number and position of teeth missing due to oral disease. These findings suggest that the quantity of teeth lost does not necessarily reflect the impact of tooth mortality on OHRQoL and that future studies should take this into consideration.

## Background

In a recent systematic review it was concluded that tooth loss impacts on quality of life [1] independently of the instrument used to measure quality of life or the social context. Studies have shown that the absolute number of teeth as well as their relative position in the mouth are associated with impairment of Oral Health-Related Quality of Life (OHRQoL) [1]. However, it is unusual to classify tooth loss in order to produce information on both the number and position of those teeth in the same measure.

While many countries have seen a decrease in the prevalence of tooth loss, this oral condition still represents a significant health concern among Brazilian adults [2] and in 2010, in the Global Burden Disease study, severe tooth loss was pointed as one of the 100 health conditions causing injuries among populations [3]. In Brazil many factors have been found to be associated with tooth loss, including socio-economic, demographic, dental care use, personal characteristics and clinical oral health status [4,5]. These contextual variables may also affect the subjective perception of oral health and its impacts on quality of life (QoL). Thus, it is important that the impact of tooth loss on OHRQoL be analyzed by means of conceptual theoretical models, such as those of Andersen & Davison (6), which respect the influence of sex, age, income and health practices on health outcomes and consequently on OHRQoL [6,7]. These personal conditions can act as effect modifiers and interfere with the perceived impact on quality of life (QoL) with the onset of clinical conditions [8].

Recently, patient-based outcome measures have extensively been collected in epidemiologic studies and in national surveys [6,9–12], as it is important to obtain knowledge of what people say about their health, and how they feel about their oral health status in order to direct health strategies to provide treatment of oral diseases and rehabilitation in cases of tooth loss. These data are obtained through instruments developed to measure OHRQoL. Of all these instruments, the Oral Health Impact Profile (OHIP-14) [13] is the most widely used to assess the impact of oral health on quality life in adults and the elderly [12]. The OHIP-14 intends to assess the dysfunction, discomfort and disability caused by oral condition [10].

Measuring the impact of tooth loss on OHRQoL is a great challenge, mainly because in epidemiologic surveys, only quantitative data, expressing the number of missing teeth are available. In the literature, information about the position in which teeth were in the mouth has hardly been explored, and there is no measure that combines number and position of missing teeth, to enable analysis of the impact of tooth loss. In our study, a tooth loss classification was created and applied in an adult population in order to examine whether it is the number of teeth or the position of the tooth lost, or both that have greater impact on oral health-related quality of life. Our hypothesis was that the effect of tooth mortality on oral health related quality of life is underestimated if these significant factors are not accounted for.

## Methods

### Study design and location

This was a cross-sectional study conducted in the city of Piracicaba, São Paulo State, Brazil, with a household probability sample. In 2010, the population of Piracicaba consisted of a total of 368,836 residents, and in the urban area, the adult population aged 20–64 years old was 170,611 [14].

### Sample selection

For the purpose of this study only adults residing in Piracicaba, aged 20 to 64 years old, were eligible to participate. The sample size was calculated, in order to obtain a representative sample of the adult population of this municipality. The prevalence of caries experience in adults [15], adjusted for the Piracicaba population size for adults aged 20 to 44 years old, and 45 to 64 years old, of 70.2% and 90.9% respectively, was the basis of the calculation. A confidence interval of 95%, an accuracy of 10% and a design effect of 1.5 were adopted. A 30% increase was added to this total in order to compensate for possible loss, thereby resulting in estimate of 172 adults 20–44 years old and 68 45–64 years old, total sample was 240 individuals. To select the houses, considering the possibility of refusals, we added 30% of this sample size which comprised 342 houses, divided by the 30 census tracts selected for the study, resulting in a fraction of 11.4 houses per census tract.

Sample selection was carried out in two stages. In the first stage, the unit of selection was the census tract and from 456 census tracts, 30 were randomly selected (plus 2 in case substitutions were needed). The second stage consisted of the selection of households, and a 30% increase in the probabilistic sample size to select the houses was used to compensate for non-responses. This resulted in a total of 342 houses, divided by the 30 census tracts selected for the study, resulting in a fraction of 11.4 houses per census tract. Based on the average population size of each census tract, 11 houses per tract were randomly selected and then 1 adult per house was also randomly selected. The inclusion criteria were being a Piracicaba resident aged 20-64-years-old, with the mental capacity to answer the study questionnaire and agreeing to participate in the research. In order to minimize non-response, the examiner returned to each house up to three times in cases of absenteeism.

### Data collection

Data collection was performed between June 2011 and September 2011, by one dentist (the examiner) and a community health agent. Oral health examinations and interviews to complete the questionnaires were carried out at the participants’ homes.

The examiner was trained by an experienced examiner, including theoretical and practical discussions for approximately eight hours in order to obtain at least 90% agreement for coronal caries and periodontal status [16]. Intra-observer agreement ranged from 96.5 to 100% for caries and periodontal disease. Kappa values were high at 0.89–1.00 and within the standards of reliability [17].

Caries experience (DMFT index), periodontal disease (Community Periodontal Index -CPI), presence of biofilm [18], treatment needs for caries, and use and/or need of dental prosthesis were the conditions evaluated according to WHO criteria using WHO probe and mirror [16]. Clinical exams performed in the participants’ homes, with subjects seated on a regular chair. A questionnaire was applied to obtain demographic, socioeconomic, health literacy [19], use of dental services data and OHRQoL. The instrument used to assess OHRQoL was the OHIP-14 [13], which was validated in Brazil by Oliveira & Nadanovisk [20]. An interview was held after the oral examinations and was also conducted by the examiner. The questionnaire contained a total of 86 questions, part of these were derived from the National Epidemiological Survey of Oral Health carried out in Brazil, 2011 [21], while the others, obtained from different sources, were pilot tested before being used in the study. In the pilot test, adults (n = 10) aged 20–64 years old, were interviewed by the examiner in order to check their understanding of the questionnaire, and how long it would take to interview each person.

### Data analysis

Data were analyzed using the Statistical Package for the Social Sciences (SPSS), version 19.0 software program. Descriptive weighted analyses were performed to obtain the frequency, mean, median, and standard deviation (SD) of variables which were the clinical conditions examined. The weighted analyses were performed according to Levi & Lemershow [22], considering the probability of the individual being randomly selected. The outcome for this study was OHRQoL, measured by OHIP severity, *i.e.*, the total OHIP-14 score (sum of the Likert-type responses for the fourteen questions, range 0–56), and OHIP prevalence, *i.e.*, the relative frequency of one or more impacts “often/very often” on OHQoL [9].

**Figure 1:**
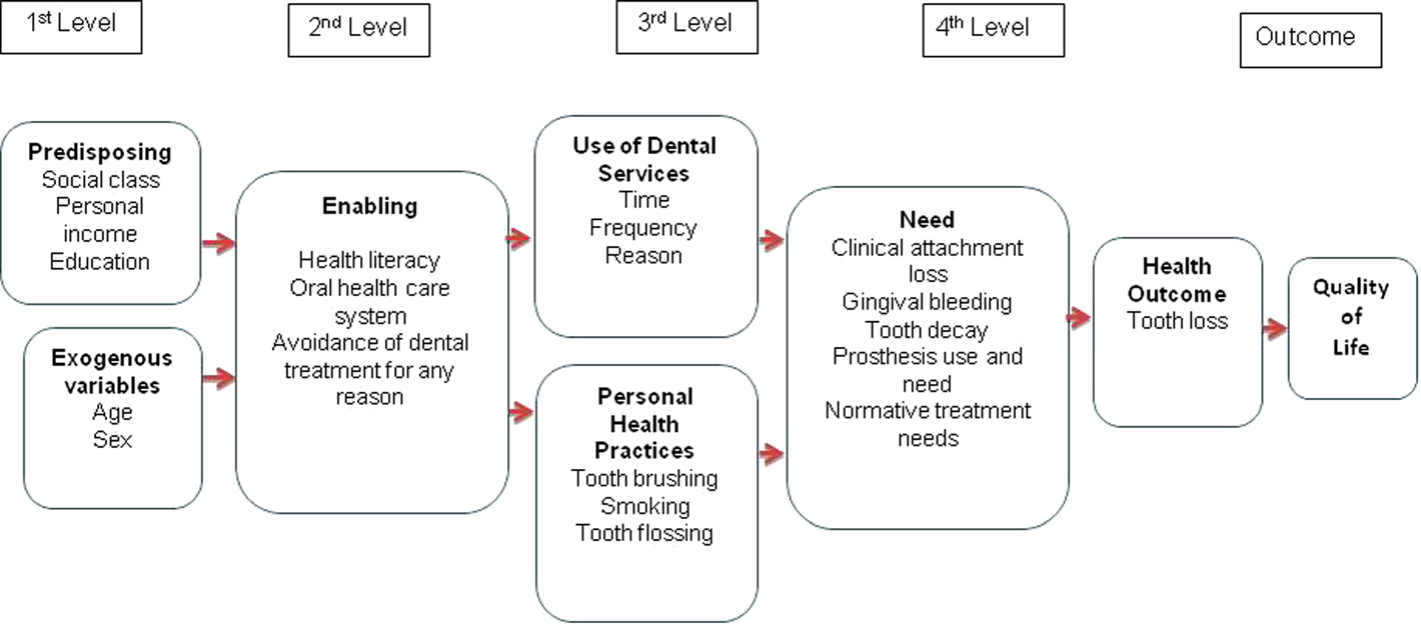
**Conceptual framework for oral health-related qualify of life adopted for the study.**

The independent variables studied were selected according to a validated conceptual framework [6] adapted for the study (Figure 1). The Andersen & Davidson model [6] considered the socioeconomic context of individuals, assessed demographic characteristics, dental care use and personal habits, as well as clinical status, and it seemed to explain the role played by tooth loss on OHRQoL better when evaluated by a factorial equation [8]. After a descriptive analyses the variables selected were categorized and/or dichotomized for statistical analysis.

In the first level, at exogenous block age, the sample was divided into two groups: 20 to 44 years and 45 to 64 years; and sex was considered male and female. In the predisposing block, personal income was considered high [more than 2 minimum wages (MW)], medium (1 to 2 MW) and low income (up to 1 MW) according to Brazilian standards (21). The MW was equivalent to U$250 at the time the data collection. Education was classified into three groups, namely up to 4 years of schooling (elementary), 5 to 11 years (basic education) of schooling, and over 11 years (more than high school level). Social class was classified according to the criteria of Graciano *et al.* [23], which consider family income, number of residents per house, home ownership or rented house, adult occupation and adult education as factors that influence inclusion in a social class classification. A score was attributed to each criterion, and afterwards the sum of the scores provided the total results, which were classified into six conditions within the class (?). Better conditions, such as higher income, and higher number of years of schooling; lower number of residences per house; non manual occupation of the participant and being the owner of a house, represented higher social class. The variable social *class(?)* was grouped into three categories, lower, high lower and middle class(?) or more, to achieve the best distribution of each category.

In the second level, the enabling block, oral health care was considered to be the service used in the last dental visit, categorized as public or private (covered out of pocket or by health insurance). Avoidance of dental treatment for any reason was classified as “yes” or “no”. Health literacy was assessed through five questions adapted from Ishikawa et al. [19], but for the purpose of regression analyses only two items that met p < 0.20, were selected; “feel able to collect oral health related information” and “feel able to judge oral health information” with their answers dichotomized into agree and do not agree.

In the third level, the use of dental services block, time of the last appointment was stratified into less than one year, between one and two years or three years or more; the reason for visiting a dentist was grouped into routine, pain or other needs; the frequency of dental services use was dichotomized into regularly or for emergency only. As regards personal health practices, tooth brushing frequency was categorized as brushing their teeth 2 or less times per day and 3 or more times/day; tooth flossing was classified as yes or no, and smoking habit also was classified as yes or no.

In the fourth level, oral health needs were evaluated according to clinical status measures, with caries being evaluated as presence of one or more decayed teeth, periodontal clinical attachment loss (CAL) of 4 mm or more; presence or absence of gingival bleeding; presence of normative treatment needs; use and need of fixed or removable dental prosthesis was measured considering yes or no, respectively.

Tooth loss was the main explanatory variable and was measured using an ordinal scale, based on tooth position and number of missing teeth, which was developed specifically for this study. In the clinical exam we evaluated the reason for tooth loss, if it was due to oral disease (caries and periodontal disease) or due to orthodontic and others reasons. For the classification, our study considered tooth loss due to oral disease. The tooth loss classification was:

- 0: No tooth lost due to caries or periodontal disease.
- 1: loss of 1 to 4 first permanent molars.
- 2: loss of up to 12 posterior teeth, excluding subjects who had lost only the first permanent molars.
- 3: loss of up to 12 teeth including an anterior tooth.
- 4: loss of more than 12 teeth (13–31).
- 5: edentulous.

The tooth loss classification was created after a literature review of the distribution of missing teeth among adults. This classification intends to measure tooth loss functionally and esthetically considering qualitative data (position of missing teeth) and quantitative data (number of missing teeth). Category 1 was considered, because findings that have shown that the tooth most frequent missing is the first molar [4,15]. The cut-off in twelve missing teeth for the categories 2, 3 and 4, was based on short arch [24]. Tooth loss classification did not take into account the number of teeth present, but number of missing teeth with prosthetic space. Thus, those who had no teeth missing teeth due to oral disease were considered fully dentate, even if they did not have 32 teeth.

Bivariate and multivariate analyses were performed depending on the outcome variable. For the OHIP with severity as the outcome, a log-negative binomial regression was used to produce the final model according to the four levels of a hierarchical approach (Figure 1). For OHIP prevalence, we used logistic regression in order to produce the final model. The modeling process was performed by step-wise method in each level. The exponential of β regression coefficients were interpreted as Prevalence Rate Ratio (PRRs) and Odds Ratio (OR), respectively. For the hierarchical analyses the independent variables were adjusted at each block after bivariate analysis, considering the theoretical framework. Variables with p < 0.20 fit the subsequent block, from first to fourth resulting in the final model. Statistical significance was set at the 5% level (two-tailed tests). The regression analyses were performed for complex samples, using a weight factor according to Levi & Lemershow [22].

### Ethics

The protocol for this study was approved by the Research Ethics Committee of Piracicaba Dental School, University of Campinas. All adults who participated in the study signed a consent form.

## Results

**Figure 2:**
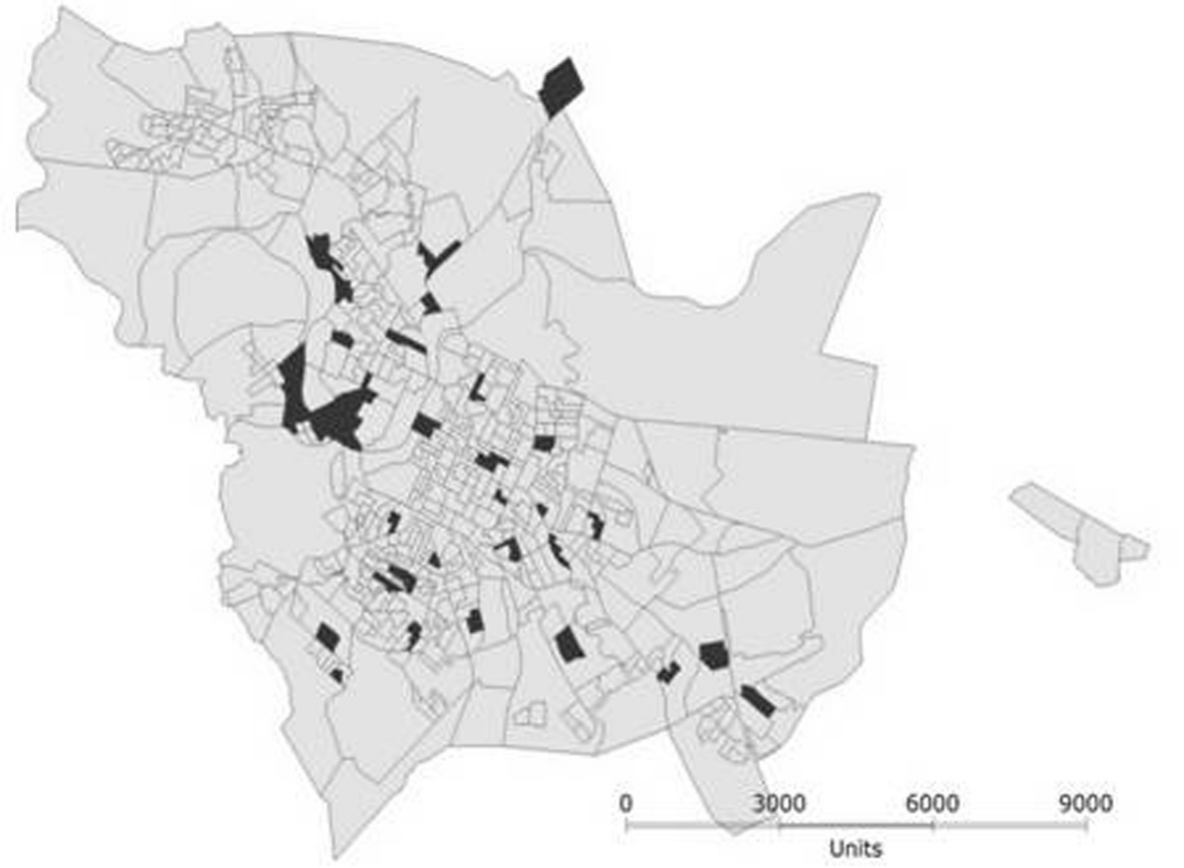
**Randomly selected census tracts on map of Piracicaba.**

A total of 248 adults participated in the study, representing an estimated 149,635 adults aged 20 to 64 years old residents of Piracicaba among the population in the same age range (170,611). Figure 2 illustrates the 32 census tracts randomly selected for the study, which were well distributed across the city.

Two-thirds of the participants were female, 30.9% were 20–44 years old (mean age = 41.98 ± 12.67), 78.2% were White. With respect to socioeconomic factors, 38.7% had a lower personal income, 28.5% had attended school for 4 years or less and 13.3% belonged to the low social class. Other sample characteristics are shown in Table 1.

**Table 1:** **Demographic and socioeconomic characteristics of 20–64 year-old residents in Piracicaba, SP, Brazil, 2011**

**Sample characteristics**			**Total**	**Weighted**	**IC 95%**
			**% (n)**	**% (n)**	**%**
**Demographic**	***Gender***	Male	27.8(69)	33.5 (50166,438)	22.6- 46.6
		Female	72.2 (179)	66.5 (99468,883)	53.4- 77.4
	***Age (years)***	20–44	55.6 (138)	30.9 (46205,521)	23.3- 39.3
		45–64	44.4 (110)	69.1 (103429,800)	60.4- 76.7
	***Race***	White	79.8 (198)	78.2 (117034,328)	67.1- 86,3
		Black	8.5 (21)	6.6 (9886,771)	3.4- 12.4
		Mixed race	10.9 (27)	14.8 (22093,398)	6.9- 28.8
		Asian	0.8 (2)	0.4 (620,833)	0.1- 1.8
**Socioeconomic**	***Marital status***	Single	18.1 (45)	12.2 (18299,063)	7.1- 20.2
		Married	70.2 (174)	73 (1092223,210)	66.4- 78.7
		Divorced + widow	11.7 (29)	14.8 (22113,049)	8.6- 25.4
	***Personal income***	Up to 1 MW	38.7 (96)	38.7 (56864,195)	30.9- 47.2
		1- 2 MW	21.8 (54)	17.6 (25904,271)	12.4- 24.4
		+ 2 MW	37.9 (94)	43.7 (64181,750)	33.0- 55.0
	***Family income***	Up to 1 MW	3.6 (9)	3.2 (4718.33)	1.1- 9.0
		1- 2 MW	12.1 (30)	9.7 (14123,959)	5.4- 16.6
		+ 2 MW	81.9 (203)	87.1 (127487,081)	78.4- 92.7
	***Educational level***	Up to 4 years	17.3 (43)	28.5 (42576,751)	16.8- 43.9
		5 to 10 years	27.8 (69)	29.2 (43671,486)	23.3- 35.9
		+ 11 years	54.8 (136)	42.4 (63387,084)	28.2- 57.9
	***Social class***	Medium	17.3 (43)	11.5 (17197,083)	6.5- 19.5
		High lower	67.3 (167)	75.3 (112462,925)	65.3- 82.9
		Lower	15.3 (38)	13.3 (19975,313)	8.4- 20.5

Note: MW is minimum wage.

Variables “personal income” and “family income” did not complete 100% percentage value due to missing cases.

The OHIP prevalence was 48.1% (95% CI 41.6-54.7) and the mean OHIP severity score was 10.21 (SE 1.16) (Table 2). The OHIP-14 dimensions that were most affected were psychological discomfort, physical pain and psychological disability (Table 2).

**Table 2:** **OHRQoL stratified by OHIP-14 dimension of adults residing in Piracicaba, SP, Brazil, 2011**

**Dimension**	**Prevalence: % reporting 1+ impacts fairly/very often (CI 95%)**	**Severity:** ***mean*** **OHIP score (Std. Error)**
**Functional limitation**	10.4 (6.8-15.6)	0.89 (0.16)
**Physical pain**	19.6 (13.0-28.4)	2.10 (0.19)
**Psychological discomfort**	35.8 (28.7-43.6)	2.86 (0.18)
**Physical disability**	17.0 (11.0-25.2)	1.50 (0.24)
**Psychological disability**	19.4 (13.5-27.0)	1.58 (0.22)
**Social disability**	6.0 (3.2-10.9)	0.61 (0.20)
**Handicap**	8.4 (4.8-14.4)	0.66 (0.15)
**Total OHIP14 score**	48.1 (41.6-54.7)	10.21(1.16)

Note: The table presents weighted values.

### Tooth loss classification

**Figure 3:**
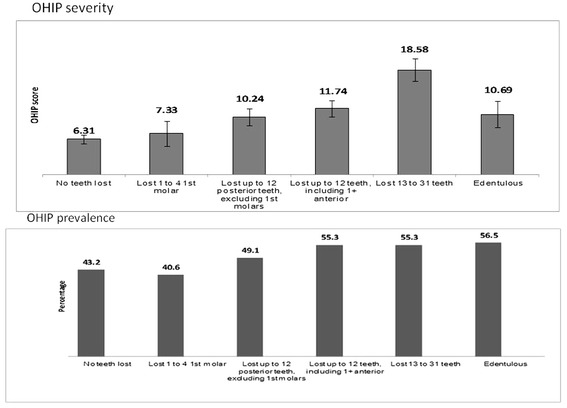
**OHRQoL (OHIP severity and prevalence) according to tooth loss classification among adult residents in Piracicaba, SP, Brazil, 2011.**

The prevalence of tooth loss was 80.5%; in other words, 19.5% (n = 70) of all participants had not lost any tooth as a consequence of oral diseases, 4.4% (n = 12) lost 1–4 first molars only, 17.8% (n = 45) lost up to 12 posterior teeth, excluding first molars, 26.8% (n = 62) lost up to 12 teeth, including one or more anterior teeth, 24.9% (n = 45) lost between 13 and 31 teeth and 6.6% (n = 13) were completely edentulous (weighted percentages). Gradients in OHRQoL increased according to the number of teeth lost and their position as per the tooth loss classification (Figure 3).

The bivariate analyses for both outcomes, namely OHIP prevalence and severity, are shown in Table 3. There were statistically significant associations between OHRQoL and socioeconomic factors, dental care use, smoking, untreated caries and oral health literacy (Table 3).

**Table 3:** **Factors associated with OHRQoL among adults residing in Piracicaba, SP, Brazil, 2011**

**Factor**			**Total N %**	**OHIP prevalence n/%**	**PR**	**p**	**OHIP severity mean (CI95%)**	**PRR**	**p**
**All**			**248 (100)**	**115 (48.1)**			**10.21 (7.85-12.57)**		
**Demographic**	*Sex*	*Male*	69 (33.5)	28 (28.3)	0.72	0.16	8.54 (3.99-13.09)	0.95	0.69
		*Female*	179 (66.5)	87 (71.7)	1		11.05 (9.42-12.68)	1	
	*Age (years)*	*45–64*	110 (69.1)	55 (71.5)	1.30	0.19	10.19 (6.59-13.79)	1.18	0.19
		*20–44*	138 (30.9)	60 (28.5)	1		10.25 (8.22-12.28)	1	
**Socioeconomic**	*Social Class*	*Lower*	38 (13.3)	23 (15.9)	3.54	0.02	14.75 (11.53-17.96)	2.21	<0.01
		*High Lower*	167 (75.2)	79 (76.5)	2.07		10.0 (6.83-13.17)	1.58	
		*Medium*	43 (11.5)	13 (7.6)	1		6.31 (4.78-7.84)	1	
	*Education level*	*Up 4 years*	43 (28.5)	25 (29.6)	2.39	<0.01	9.74 (3.53-15.96)	1.49	<0.01
		*5-11 years*	69 (29.2)	40 (34.8)	2.37		11.92 (8.52-15.32)	1.61	
		*+ 11 years*	136 (42.4)	50 (35.6)	1		9.34 (7.53-11.16)	1	
	*Personal income*	*Up to 1 MS*	96 (38.7)	59 (52.3)	2.95	<0.01	14.23 (10.81-17.66)	1.84	<0.01
		*1- 2 MS*	54 (17.6)	23 (15.9)	1.37		11.12 (6.64-15.59)	1.30	
		*+ 2 MS*	94 (43.7)	33 (31.8)	1		6.66 (4.09-9.23)	1	
**Use of dental care**	*Service used*	*Public*	55 (19.0)	35 (25.2)	2.01	<0.01	16.42 (13.27-19.58)	1.55	<0.01
		*Insurance*	58 (27.0)	18 (19.4)	0.52		6.58 (3.81-9.34)	0.74	
		*Private*	131 (54.0)	61 (55.4)	1		9.91 (7.34-12.48)	1	
	*Frequency of dental visits*	*Emergency*	132 (52.4)	73 (55.3)	2.26	<0.01	13.17 (10.81-15.53)	1.81	<0.01
		*Regular*	113 (47.6)	40 (41.3)	1		6.87 (4.77-8.97)	1	
	*Reason for dental care Use*	*Pain*	55 (20.6)	32 (26.7)	2.75	<0.01	15.47 (11.39-19.55)	1.93	<0.01
		*Need*	54 (24.7)	33 (33.5)	3.11		13.75 (9.72-17.77)	1.94	
		*Routine*	131 (54.7)	44 (39.8)	1		6.55 (4.42-8.68)	1	
**Health behaviour**	*Tooth Brushing*	*≤2 per day*	89 (35.9)	48 (44.9)	0.62	0.08	13.40 (10.24-16.56)	1.35	0.04
		*>2 per day*	159 (64.1)	67 (55.1)	1		8.42 (6.07-10.78)	1	
	*Tooth flossing*	*unusual*	148 (61.2)	75 (64.6)	1.54	0.01	10.56 (7.68-13.44)	1.15	0.35
		*usual*	100 (38.8)	40 (35.4)	1		9.66 (6.49-12.82)	1	
	*Smoking*	*Yes*	48 (23.2)	30 (27.0)	2.26	0.01	13.32 (10.81-15.53)	1.43	0.04
		*No*	200 (76.8)	85 (73.0)	1		9.27 (7.31-11.22)	1	
**Clinical conditions**	*Decayed Teeth*	*Yes*	91 (35.4)	62 (52.9)	4.78	<0.001	15.16 (12.09-18.23)	2.02	<0.001
		*No*	157 (64.6)	53 (47.1)	1		7.50 (5.05-9.96)	1	
	*CAL>4mm*	*Yes*	82 (43.0)	40 (45.6)	0.59	0.59	10.03(5.72-14.33)	1.21	0.22
		*No*	166 (66.9)	75 (54.4)	1		10.35 (8.78-11.91)	1	
**Health literacy**	*feel able to collect oral health information*	*Do not agree*	106 (34.3)	48 (39.4)	1.73	0.04	13.32 (9.94-16.70)	1.27	0.04
		*Agree*	142 (65.7)	67 (60.6)	1		8.58 (6.09-11.08)	1	
	*feel able to judge oral health information*	*Do not agree*	103 (48.0)	56 (57.2)	1.06	<0.001	10.74 (7.71-11.73)	1.01	<0.001
		*Agree*	145 (52.0)	59 (42.8)	1		9.72 (7.12-14.36)	1	
**Tooth loss**	*Edentulous*		13 (6.6)	7 (56.5)	2.38	0.16	10.63 (5.5-15.7)	1.80	0.02
	*Lost 13–31 teeth*		45 (24.9)	29 (55.3)	3.70	0.001	16.05 (10.5-21.6)	2.71	<0.001
	*Loss of up to 12 teeth, including 1+ anterior*		62 (26.8)	32 (44.0)	2.18	0.03	9.73 (6.3-13.1)	1.65	0.01
	*Loss of up to 12 posterior teeth, excluding 1* ^*st*^ *molars*		45 (17.8)	20 (49.1)	1.64	0.21	8.09 (5.5-10.7)	1.37	0.14
	*Loss of 1–4 1* ^*st*^ *molar*		12 (4.4)	4 (40.6)	1.02	0.97	7.12 (2.8-11.4)	1.20	0.57
	*No teeth lost*		70 (19.5)	23 (43.2)	1		5.92(4.6-10,2)	1	

Note: The table presents weighted values.

*Significance level is p value<0,05.*

Those who had lost up to 12 teeth, including 1+ anterior teeth; those who had lost 13–31 teeth, and the edentulous, compared with fully dentate adults presented higher scores for the impact of oral health on quality of life. Adults who presented caries and used dental care services due to dental pain or treatment needs were more likely to have higher OHIP scores. The OHIP prevalence analysis did not yield significant odds ratios for tooth loss. Those who presented decayed teeth; used dental services due to pain and those with lower personal income were more likely to have one or more impacts on OHRQoL (Table 4).

**Table 4:** **Multivariate analyses results evaluating negative impact on OHRQoL among adults residing in Piracicaba, SP, Brazil, 2011**

**Risk indicator**		***OHIP prevalence***			***OHIP severity***		
		**OR adjusted**	**CI (95%)**	**p**	**PRR adjusted**	**CI (95%)**	**p**
**Tooth Loss**	Edentulous	3.92	0.94-16.89	0.061	2.66	1.55,4.57	<0.001
	*Loss of* 13 to 31 teeth	1.08	0.38-3.05	0.886	2.33	1.49,3.63	<0.001
	*Loss of* up to 12 teeth, including 1+ anterior	0.96	0.37-2.51	0.939	1.63	1.06,2.51	0.026
	*Loss of* up to 12 posterior teeth, excluding 1^st^ molars	1.37	0.38-4.97	0.626	1.44	0.95,2.18	0.088
	*Loss of* 1 to 4 1^st^ molar	1.28	0.30-5.52	0.738	1.23	0.71,2.15	0.463
	No teeth lost	1			1		
**Caries**	Yes	3.96	1.85-8.51	<0.001	1.57	1.09,2.26	0.015
	No	1			1		
**Reason for use of dental services**	Need	1.94	0.82-4,61	0.132	1.84	1.24,2.71	0.014
	Pain	2.43	1.01-5.82	0.047	1.67	1.11,2.51	0.002
	Routine	1			1		
**Personal income**	Up to 1 MS	2.80	1.26-6.42	0.015			
	1- 2 MS	0.91	0.39-2.46	0.969			
	+ 2 MS	1					

Note: The table presents weighted values.

*Significance level is p value<0,05.*

## Discussion

Previous studies have demonstrated the impact of tooth loss on OHRQoL [1,9,25,26], but identifying how this impact occurs is a challenge to epidemiologic studies. In a systematic review, the tooth loss impact on OHRQoL was found to be related to the number of missing teeth and position (anterior or posterior) of the missing teeth. However, these studies showed the impact of tooth loss on OHRQoL according to the number of teeth and position of teeth separately [1]. The present study presents a classification of tooth loss that may be a useful indicator of the number and position of the missing tooth. The ordinal classification for tooth loss used in this study allows for measuring the number of teeth lost and their position in the same variable. We observed that the severity of impact on OHRQoL was higher when the number of teeth lost was above 13, however we also found that when tooth loss was up to 12 teeth, including any anterior missing teeth, this also had a severe impact on OHRQoL, when compared with fully dentate adults.

The results of this study showed the importance of assessing the impact of tooth loss on OHRQoL measuring tooth loss quantitatively (number of missing teeth) and qualitatively, including the position of missing teeth in the tooth loss indicator. This classification allowed differentiating the perception of OHRQoL impacts even when the number of missing teeth was the same. Those who had up to twelve missing teeth including anterior teeth were more likely to present higher scores on OHIP than those who had up to twelve missing, but only posterior teeth.

To extend the evaluation of subjective outcomes such as OHRQoL, we analyzed the dependent variable in two ways. First, as a categorical outcome, which was the prevalence of oral impacts fairly/very often, and second, as a numerical outcome. This enlargement of the outcome evaluation works to improve the interpretation of OHRQoL, according to the recommendation by Tsakos *et al.* [7], to use more than one method to analyze “participant-based outcomes” that are measured by means of scores. Our study followed their recommendation and performed both regression models; prevalence and severity of OHIP-14. By using this approach, we recovered data missed by dichotomization of scores and found the variables associated with the prevalence of severe impacts that were reported “fairly/very often”, as well with the total scores.

The two analyses performed in this study showed different results for tooth loss. Tooth loss was associated with the gradients of OHIP severity according to the number and position of the lost teeth as illustrate in Figure 3. Those who lost up to twelve teeth, including one or more anterior teeth; those who lost from thirteen to thirty one teeth and the edentulous presented more impact on OHRQoL when compared with fully dentate adults. Lathi *et al.* [27] found that impaired subjective oral health was more often reported for those with fewer natural teeth and our findings concur. Studies that consider functional dentition, counting 20 teeth or more, should pay attention to the position of the teeth present, because even among participants in this study who had more than twenty teeth, the position of the lost teeth had an impact on OHRQoL. However, no tooth loss indicator that considered the number and position of teeth in the same measure, was found in the literature.

Among the individuals who had up to twelve missing teeth, the position (anterior or posterior) appeared to interfere with severity of OHIP. This fact may show the importance of esthetics and appearance related to tooth loss. It is Interesting that our findings of the impact of oral health on psychological discomfort among the adults of Piracicaba is considerably high in comparison with adults from Canada, New Zealand and Australia [9]. Personality profiles may influence dental perceptions and OHRQoL impacts as well, a fact that is a characteristic of this population, since subjective measures of OHRQoL and cultural aspects play an important role in the quality of life values [28]. Further studies considering the tooth loss classification should be conducted in other countries, which may show different results.

The prevalence of severe impacts (fairly/very often) was associated with untreated caries, the reason to use dental care, and personal income. Untreated caries, severe tooth loss and periodontitis were pointed out as being among the 100 conditions that cause burden in the Global Burden Disease [3].

We need to mention that socioeconomic factors may affect the impact on OHRQoL perception; moreover, various studies have found that socioeconomically disadvantaged people have higher risks of disease and suffer from worse health conditions [9,11,26,29,30]. Our study found similar results, with low personal income related to the prevalence of impacts on OHRQoL. Poverty and inequalities in health are known causes of disease [31] and low income interferes with the perception of life control and stress that affect self-perception of oral health [30]. This was the case in our study in which, even after adjusting for missing teeth, low family income was significant. Thus, material deprivation or low income has a close relationship with oral diseases, including both the condition of tooth loss and the impact on quality of life. Finding a solution to the problem of health inequalities is a significant challenge [29] but it is fundamental that strategies are implemented to reduce the disease burden and improve access to care.

This paper does not intend to infer cause-and-effect by means of a cross-sectional study, despite the fact that it was a population-based study. Mental capacity was not investigated in this study. However, the adults needed to present normal cognitive capacity to be able to answer the questions put to them. Moreover, the adults were examined before the questionnaire was applied, which may cause a bias of information, but the examiner did not inform the participants of their oral health status at this time. The tooth loss classification, however, can represent a useful tool for evaluation for future epidemiologic studies. The findings of this study are different from those of others because of the nature of subjective and multidimensional aspects of patient-based outcomes that are culturally rooted [7]. Studies based on patient perception are important to improve knowledge about the impact on OHRQoL, because adults and children lose many hours of work and study annually owing to the problems and discomfort associated with oral diseases [32].

The use of OHRQoL measurements, when associated with clinical indicators in a dental public health context, can be useful for planning public health services and health promotion strategies among adults who are most in need. Patient-based outcomes highlight the need for public health interventions to reduce health inequalities, as higher scores on OHRQoL were found among those who paid a fee for dental services [33]. Strategies that increase the opportunities for health care for those who present impacts on OHRQoL, combined with a preventive approach based on common risk and tooth loss rehabilitation, could help to diminish inequities among adult populations and improve quality of life.

## Conclusion

Our analyses showed oral health quality of life gradients consistent with the number and position of teeth missing due to oral disease, as measured by the OHIP-14 severity index, when controlling for presence of untreated caries and the reason for using dental services. These findings suggest that the mere quantity of teeth lost does not necessarily reflect the true impact of tooth mortality on OHRQoL and that future studies should take this point into consideration. These findings should also be considered in oral health promotion strategies targeting adults in Brazil and elsewhere.
